# W342F Mutation in CCaMK Enhances Its Affinity to Calmodulin But Compromises Its Role in Supporting Root Nodule Symbiosis in *Medicago truncatula*

**DOI:** 10.3389/fpls.2017.01921

**Published:** 2017-11-16

**Authors:** Edgard Jauregui, Liqun Du, Cynthia Gleason, B. W. Poovaiah

**Affiliations:** ^1^Laboratory of Molecular Plant Science, Department of Horticulture, Washington State University, Pullman, WA, United States; ^2^College of Life and Environmental Sciences, Hangzhou Normal University, Hangzhou, China; ^3^Department of Plant Pathology, Washington State University, Pullman, WA, United States

**Keywords:** Ca^2+^/calmodulin-dependent protein kinase, calcium, calmodulin, symbiosis, *Medicago truncatula*

## Abstract

The calcium/calmodulin-dependent protein kinase (CCaMK) is regulated by free Ca^2+^ and Ca^2+^-loaded calmodulin. This dual binding is believed to be involved in its regulation and associated physiological functions, although direct experimental evidence for this is lacking. Here we document that site-directed mutations in the calmodulin-binding domain of CCaMK alters its binding capacity to calmodulin, providing an effective approach to study how calmodulin regulates CCaMK in terms of kinase activity and regulation of rhizobial symbiosis in *Medicago truncatula*. We observed that mutating the tryptophan at position 342 to phenylalanine (W342F) markedly increased the calmodulin-binding capability of the mutant. The mutant CCaMK underwent autophosphorylation and catalyzed substrate phosphorylation in the absence of calcium and calmodulin. When the mutant W342F was expressed in *ccamk-1* roots, the transgenic roots exhibited an altered nodulation phenotype. These results indicate that altering the calmodulin-binding domain of CCaMK could generate a constitutively activated kinase with a negative role in the physiological function of CCaMK.

## Introduction

Calcium/calmodulin (Ca^2+^/CaM)-mediated signaling plays important roles in sensing and transducing environmental stimuli and developmental cues (Du and Poovaiah, 2005; Poovaiah et al., 2013), plant immune responses (Du et al., 2009) and mutualistic plant–microbe interactions (Gleason et al., 2006; Yuan et al., 2017). One important effector protein of this Ca^2+^/CaM-mediated signaling in plants is the Ca^2+^/CaM-dependent protein kinase, CCaMK (Patil et al., 1995). CCaMK is nuclear localized and lies downstream of the cellular calcium spiking response that occurs in the symbiosis pathway. After activation by Ca^2+^/CaM, CCaMK phosphorylates CYCLOPS or IPD3 in *Medicago truncatula*, which are required for symbiosis (Yano et al., 2008).

CCaMK has been well studied not only because of its unique protein structure, but also due to its critical role in the symbiotic interaction between leguminous plants and bacteria and/or fungi (Patil et al., 1995; Ramachandiran et al., 1997; Harper et al., 2004). The protein structure of CCaMK contains a catalytic domain followed by a CaM-binding/autoinhibitory domain and three EF hand motifs (Sathyanarayanan et al., 2001; Sathyanarayanan and Poovaiah, 2002; Singh and Parniske, 2012). CCaMK is regulated by free Ca^2+^ which binds to the EF hand motifs and Ca^2+^-loaded CaM, which binds to the autoinhibitory domain (Lévy et al., 2004; Tirichine et al., 2006; Pumplin et al., 2010). This two-step regulatory mechanism allows CCaMK to become active and promote phosphorylation of target proteins that regulate root nodule symbiosis and mycorrhizal arbuscule development (Wang and Poovaiah, 1999; Sathyanarayanan and Poovaiah, 2002; Parniske, 2004). Both regulatory domains are important during symbiotic response (Tirichine et al., 2006; Pumplin et al., 2010). Previous reports demonstrated that expressing only the kinase domain (KD) of this protein or expressing a mutant of the threonine at position 271 (Thr271), located in the KD, resulted in the formation of spontaneous nodules in the absence of bacteria in *Medicago* roots (Sathyanarayanan et al., 2001; Gleason et al., 2006; Tirichine et al., 2006). This indicates that CCaMK’s autoinhibitory domain and the EF-hand motifs negatively regulate the kinase activity. Another report has also shown the importance of the EF hand motifs in negative regulation of CCaMK. This study indicated that the EF hand motifs bind free Ca^2+^and keep the CCaMK inactive at basal Ca^2+^ concentrations (Shimoda et al., 2012). Conversely, if free Ca^2+^ is blocked from binding to the EF hand motifs, CCaMK becomes auto-activated (Hayashi et al., 2010; Miller et al., 2013). Recent reports have demonstrated that CCaMK might be regulated by DELLA proteins (Jin et al., 2016) and/or TOR (the target of rapamycin) protein kinase (Nanjareddy et al., 2016) which are essential for symbiotic rhizobial pathway. Furthermore, CCaMK gene is very well conserved among phytozome species (Wang et al., 2015). CCaMKs have been found in peanuts (*Arachis hypogaea*) and tomato (*Solanum lycopersicum*), where it may play roles in the symbiotic pathway (Peng et al., 2017) and/or disease resistance (Wang et al., 2015).

Generally, the key to the activation of the kinase requires the release of the protein’s own autoinhibition, which can be accomplished by the binding of CaM to the autoinhibitory/CaM-binding domain (Hayashi et al., 2010; Antolín-Llovera et al., 2012). In 2011, researchers using an auto activated form of CCaMK concluded that CaM-binding is essential for nodulation development during rhizobial symbiosis, but it is not required for fungal symbiosis (Horváth et al., 2011). However, since this auto-activated form could no longer interact with either CaM or free Ca^2+^, it is critical to study the relevance of the CaM-binding alone during the activation of CCaMK (Takeda et al., 2012). Furthermore, CCaMK protein structure is unique to plants and understanding of its activation and regulation is far from clear. There are reports which have focused in its KD and visinin-like domain and have determined their importance of specific amino acids that regulate its kinase activity in addition to calmodulin and Ca^2+^ binding. Studies on the relationship between calmodulin and Ca^2+^ binding and kinase activity could reveal how this kinase interprets the Ca^2+^ signature during the establishment of plant–microbe symbioses.

In this study, we used a site-directed mutagenesis approach to alter the Calmodulin-binding/autoinhibitory domain of CCaMK in order to observe any variation of the CaM-binding capacity of this protein kinase in the presence of Ca^2+^. We generated a series of mutations at a critical residue in the autoinhibitory domain (Trp-342) and measured the effects of the mutations on CaM-binding capacity. We found that the mutant W342F has increased CaM-binding capacity, even in the absence of Ca^2+^. Interestingly, roots expressing W342F developed nodules after rhizobial inoculation, but half of these nodules were smaller than normal and were poorly colonized by bacteria.

## Materials and Methods

### Site-Directed Mutagenesis

Site-directed mutagenesis of CCaMK was performed using a high fidelity KOD DNA polymerase enzyme to construct the desired plasmid with the mutations, following protocol as described (Liu and Naismith, 2008). The mutated CCaMK cds were confirmed by sequencing.

### Expression and Purification of CCaMK and Site-Directed Mutants

The full-length of CCaMK and its site-directed mutants were cloned into the bacterial expression system pET28b and transformed into *Escherichia coli* strain BL21 (DE3)/pLysS. The bacteria carrying the above plasmids were grown in LB liquid media containing kanamycin at 37°C until OD_600_ of the culture reached 0.5 units. Once the liquid culture reached this optimal density, 0.5 mM isopropyl β-D-1-thiogalactopyranoside (IPTG) was added to induce the recombinant protein. After 3-h induction, cells were harvested and broken using lysozyme treatment (1 mg/ml) followed by sonication. The recombinant protein was purified with Ni-NTA agarose affinity beads (Qiagen) as described in the manufacturer’s manual. The purified proteins were dialyzed against buffer containing 40 mM Tris pH 7.6, 1 mM dithiothreitol (DTT), 1 mM EDTA, and 10% ethylene glycol. Dialyzed proteins were quantified by Bradford assay and stored at -80°C with 15% glycerol.

### CaM-Binding Assays

The CaM-2 from *Arabidopsis* conjugated with horseradish peroxidase (CaM-HRP) was used to study the CaM-binding property of CCaMK mutants. The induced proteins of CCaMK and its mutants in pET28b were separated by SDS–PAGE (15%) and transferred onto PVDF membrane. The membrane was blocked in binding buffer (10 mM Tris pH 7.5, 150 mM NaCl, 1 mM CaCl_2_) containing 5% non-fat dry milk for 1 h at room temperature, then incubated with milk containing binding buffer supplemented with AtCaM2-HRP (1:1000 dilution) for 1 h at room temperature. The membrane was then washed three times in binding buffer for 10 min each. To detect the CaM signal, the BM chemiluminescence Western blotting kit (Roche Applied Science) was used according to instructions from manufacturer.

### Autophosphorylation Assays

The autophosphorylation assay was performed in 10 μl reaction mixture using 0.4 μg of CCaMK protein and its mutated versions. The reaction buffer contained 50 mM HEPES pH 7.5, 10 mM magnesium acetate, 1 mM DTT, 10 μM ATP and 0.5 μCi/μl [γ-^32^P] ATP, in the presence of 5 mM EGTA with or without 1 μM of bovine brain CaM (Sigma); and 0.5 mM of CaCl_2_ with or without bovine CaM. Samples were incubated at 30°C for 30 min. To stop the reaction, SDS–PAGE sample buffer was added, followed by boiling the samples for 2 min. Samples were separated by a 12.5% SDS–PAGE. Protein gel was then dried and exposed to autoradiography film (Kodak).

### Substrate Phosphorylation Assays

All of the *in vitro* kinase assays used 0.4 μg of purified protein in a 10 μl total volume. The buffer contained 50 mM HEPES pH 7.5, 10 mM magnesium acetate, 1 mM DDT, 100 μM ATP, and 0.5 μCi/μl [γ-^32^P] ATP in the presence of 5 mM EGTA or 0.5 mM CaCl_2_ or 0.5 mM CaCl_2_ with 1 μM of bovine brain CaM (Sigma). In order to determine substrate phosphorylation, two micrograms of a bovine myelin basic protein (MBP) was used as substrate. Reactions were stopped by adding SDS–PAGE sample buffer and then boiled for 1 min in water bath. Subsequently, sample reactions were analyzed in SDS–PAGE and gel as dried. The difference of substrate phosphorylation patterns at different conditions was observed by exposing gel to the Kodak autoradiography film.

### Hairy Root Transformation

The generation of transgenic roots was performed using a binary vector (pDL28-DPR) containing the native promoter of CCaMK and a red fluorescent protein encoding region from *Discosoma* sp. (DsRed) driven by *Arabidopsis* ubiquitin-10 promoter. The full-length CCaMK and mutant gene (W342F) were digested with SpeI and SalI restriction enzymes and ligated into the binary vector. The verified constructs were transformed into *Agrobacterium rhizogenes* strain K599 to transform the rootless plantlets of wild-type and *ccamk-1* mutant of *M. truncatula* A17. The control groups used in this experiment were wild-type *M. truncatula* explants were transformed with the binary vector without any gene (empty vector). In the other control group, the *ccamk-1* mutant plants were introduced with the empty vector. Our experimental groups were as follows: The third and fourth group used *ccamk-1* plants which were transformed with the binary vector carrying the full-length CCaMK gene and W342F mutant gene, respectively. The seedlings were grown on buffered nodulation medium (BNM) agar plates with Kanamycin selection. After 3 weeks, the plants were screened for transgenic roots by using a Kodak imaging system (Model 4000MM) with corresponding filters for the expression of DsRed fluorescent protein. For the nodulation experiments, the plants with transgenic roots were inoculated with *Sinorhizobium meliloti* 2011 carrying a green fluorescent protein (GFP) reporter gene and transferred to sterilized growth pouches (Mega International). The inoculated seedlings were watered with 1/10 dilution of nitrogen-deprived nutrient media (BNM) and grown under 16/8-h light/dark cycle at 21°C for 28 days. At 28-days post-inoculation, nodules were counted and observed under a Leitz fluorescent stereomicroscope using optical filters for GFP. Green fluorescence verified the presence of living rhizobia inside the developed nodule.

### Confocal Microscopy

Selected nodules were analyzed by Zeta 510 Meta confocal microscope in order to determine the location of the bacteria in the nodules formed on the roots of *ccamk-1* complemented with wild-type and mutated version of CCaMK (W342F). Nodules were excised in half and were stained with Calcofluor white, a special fluorescent stain that allows observation of cell walls of tissues due to its binding to cellulose and chitin. For this fluorescent stain, we used excitation filter of 365 nm and emission filter 420 nm in 10× microscope lenses. To determine GFP, we used excitation filter 380 nm and emission filter 480 nm. Merged pictures were obtained and subsequently analyzed.

## Results

### A W342F Mutation in the CCaMK Autoinhibitory Binding Domain Can Positively Increase Its CaM-Binding Capacity

**FIGURE 1:**
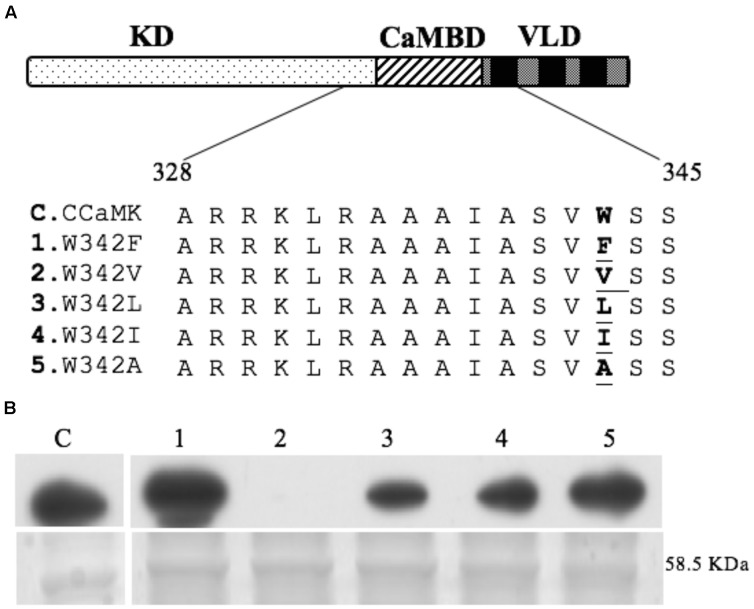
Site-directed mutants and calmodulin-binding assay. Protein structure of CCaMK contains three domains: kinase domain (KD), calmodulin-binding/autoinhibitory domain (CAMBD) and visinin-like domains (VLD). Five mutants were generated by substituting the residue Trp-432 in the calmodulin-binding/autoinhibitory domain of CCaMK **(A)**. For calmodulin-binding assay **(B)**, total proteins from *Escherichia coli* (BL21) expressing the pET28-CCaMK or its mutated versions were separated on SDS–PAGE, transferred to PVDF membrane and incubated with horseradish peroxide (HRP)-conjugated CaM in the presence of Ca^2+^ (1 mM). Upper: Calmodulin binding overlay assay of wild-type and all 5 of the mutants of CCaMK as showed in “**A**”; Lower: Coomassie staining of proteins from *E. coli* (BL21) expressing the pET28-CCaMK or its mutated versions (protein size: 58.5 kDa). The CaM-binding assay was repeated at least 4 times to corroborate the difference.

The CaM-binding domain in CCaMK interacts with CaM, therefore, site-directed mutations were generated in the CaM-binding/autoinhibitory domain to investigate whether the CaM-binding capacity and kinase activity are correlated. Four residues in the calmodulin-binding/autoinhibitory domain were selected: F327, L333, I338, and W342. Mutations in the latter one produced some interesting results. W342 was mutated to five different residues with less mass but similar hydrophobic property (**Figure 1A**). These mutants were tested for CaM-binding capability. The CaM-binding assay showed that one mutant, W342F, exhibited increased CaM-binding capacity as compared to wild-type CCaMK. W342L and W342I showed less binding to CaM in comparison to CCaMK and W342F. The mutant W343V no longer interacted with CaM, however, W342A mutant was able to bind to CaM with a similar capacity as wild-type CCaMK (**Figure 1B**).

### Significance of W342F Mutant on Its Kinase Activity

In CCaMK, the binding of CaM to the autoinhibitory domain releases its autoinhibition and activates its kinase activity. Since the W342F mutant showed an increase in CaM-binding ability, we hypothesized that the kinase activity of this mutant could be increased. Therefore, we specifically focused on this potential “gain of function” mutant, and an *in vitro* kinase assay was carried out.

**FIGURE 2:**
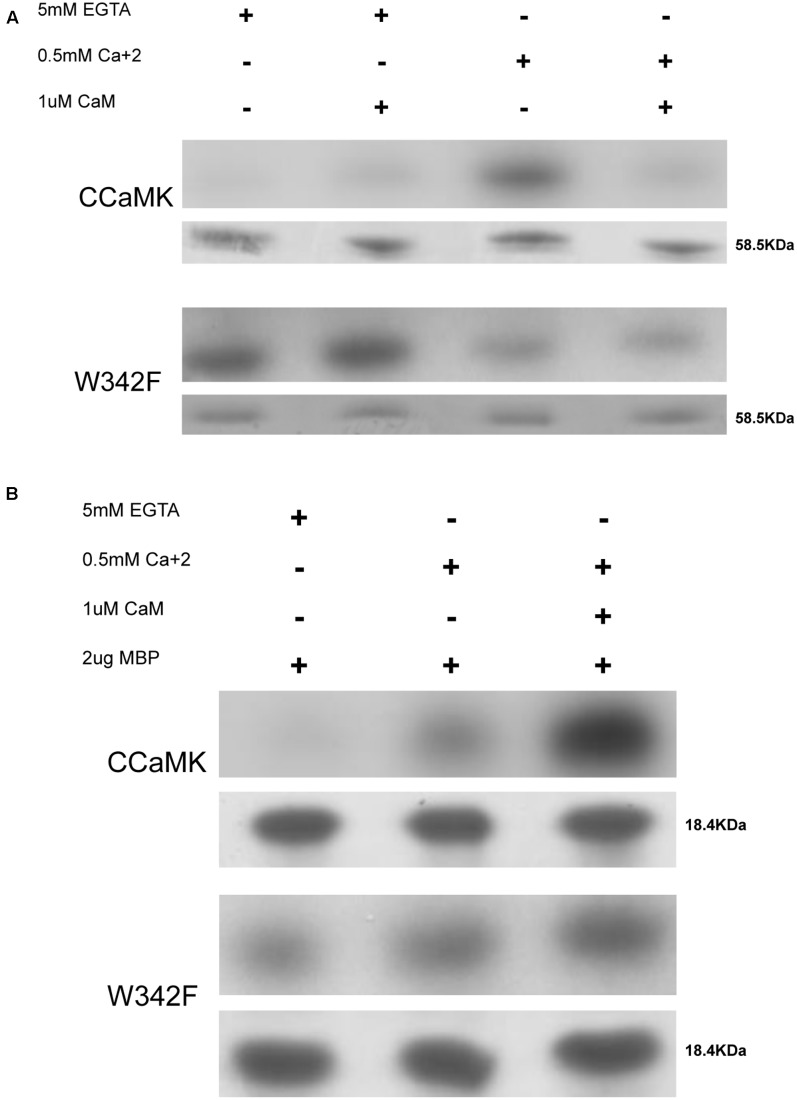
Kinase activity assays of W342F and CCaMK. Autophosphorylation was performed at 30°C for 30 min. **(A)** CCaMK did not significantly phosphorylate itself in the absence of Ca^2+^. In the presence of Ca^2+^, CCaMK showed drastically increased autophosphorylation, which remarkably decreased once calmodulin was added. The W342F mutant showed very high levels of autophosphorylation in the absence of Ca^2+^ as compared to CCaMK, indicating that the mutant behaves in a Ca^2+^-independent manner. Upper: Autoradiograph film demonstrating the autophosphorylation activity of CCaMK and W432F. Lower: Coomassie staining of the purified proteins from *E. coli* (BL21) expressing the pET28-CCaMK and W342F. **(B)** Substrate phosphorylation of CCaMK and W342F. The substrate phosphorylation of CCaMK and W342F was determined in the absence (5 mM EGTA), or presence of Ca^2+^ (0.5 mM CaCl_2_), or Ca^2+^ and calmodulin together (0.5 mM CaCl_2_ + 1 μM calmodulin). 2 μg of bovine myelin basic protein (MBP) was used as the substrate. Substrate phosphorylation of W342F in the absence and presence of Ca^2+^ did not follow a similar pattern compared to CCaMK. This mutant displayed a Ca^2+^- and/or calmodulin-independent substrate phosphorylation capacity, but the maximum level of MBP phosphorylation intensity catalyzed by W342F was remarkably lower than substrate phosphorylation intensity catalyzed by CCaMK in the presence of Ca^2+^ and calmodulin. The kinase activity assays were repeated three times. Upper: Autoradiograph film showing the substrate phosphorylation activity of CCaMK and W432F; Lower: Coomassie staining of MBP substrate (2 μg).

Normally, the autophosphorylation of CCaMK is very low in the absence of Ca^2+^, and it increases drastically once Ca^2+^ is added to the reaction mixture. Similar to previously published reports, we found that the autophosphorylation of the wild-type CCaMK was decreased after the addition of CaM (Takezawa et al., 1996; Miller et al., 2013). Interestingly we found the W342F mutant was autophosphorylated even in the absence of Ca^2+^. The W342F mutant was also autophosphorylated in the presence of CaM and 5 mM EGTA. In the presence of Ca^2+^, both wildtype CCaMK and the W342F mutant underwent autophosphorylation. However, the autophosphorylation intensity of W342F mutant was reduced in comparison to wild-type CCaMK (**Figure 2A** and Supplementary Figures 1, 2). It seems that the activation of autophosphorylation in W342F mutant is no longer dependent on Ca^2+^, on the contrary, it responds to the Ca^2+^ signal in an opposing manner as compared to wild-type CCaMK.

We used bovine MBP as substrate to test the substrate phosphorylation activities of CCaMK. These reactions were performed under three different conditions: (1) in the absence of Ca^2+^, (2) in the presence of Ca^2+^, and (3) in the presence of Ca^2+^ and CaM. The W342F mutant phosphorylated the substrate in all three conditions, with a slightly increased intensity in the presence of both Ca^2+^ and CaM. Nevertheless, in comparison to CCaMK, this mutant showed a clear reduction in substrate phosphorylation activity even in the presence of Ca^2+^ and CaM (**Figure 2B** and Supplementary Figures 1, 2). Thus, in the W342F mutant substrate phosphorylation is Ca^2+^-independent and also CaM-independent, and barely responds to Ca^2+^ and CaM inputs.

### W342F Negatively Regulates Root Nodule Symbiosis

**FIGURE 3:**
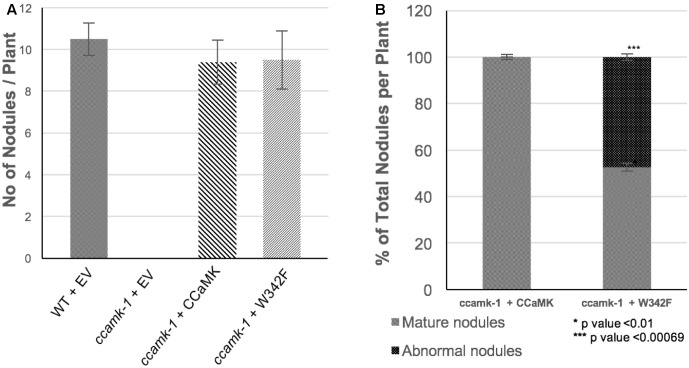
The W342F mutant plants are able to form altered nodules. The roots of *ccamk-1* mutant were transformed with the W342F mutant of CCaMK, roots of WT and *ccamk-1* transformed with empty vector were used as positive and negative controls. **(A)** The number of nodules per plant for each construct was quantitated 28-days after inoculation with *Sinorhizobium meliloti* 2011-Green fluorescent protein (GFP). **(B)** Phenotypic difference of nodule development on *ccamk-1* roots complemented with W342F and CCaMK. W342F mutation resulted in significant increase (*p*-value = 0.00069) in the number of small nodules.

**FIGURE 4:**
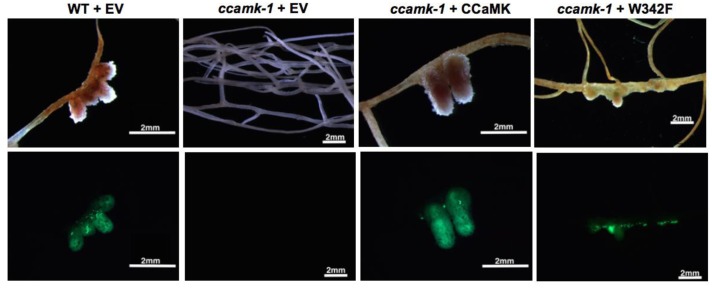
W342F mutation resulted in altered nodule development. Root phenotypes were observed for each construct. Upper: Bright field picture. Lower: GFP fluorescent image of bacteria established in the nodule structure (green color). The W342F mutant complemented *ccamk-1* for root nodule symbiosis.

With the clear indication that the phosphorylation activity of W342F mutant differs from wild-type CCaMK in its biochemical property, we wanted to test the effect of the W342F on nodule formation. The loss-of-function *ccamk-1 Medicago* plants were transformed with W342F or CCaMK by *Agrobacterium rhizogenes* transformation. The transformed roots were inoculated with *S. meliloti* 2011 tagged with a GFP. The *ccamk-1* plants inoculated with *S. meliloti* 2011-GFP did not produce nodules. The *ccamk-1* mutant complemented with wild-type CCaMK produced pink mature nodules at 28-days post-inoculation with *S. meliloti* 2011-GFP. Interestingly, at 28-days post-inoculation, the *ccamk-1* plants transformed with W342F were able to produce nodules, indicating that the W342F mutant can complement the *ccamk-1* plants (**Figures 3**, **4**). The total number of nodules produced on the roots of *ccamk-1* mutant complemented with the W342F mutant (*ccamk-1* + W342F) was comparable to the number of nodules on roots of *ccamk-1* mutant complemented with wild-type CCaMK (*ccamk-1* + CCaMK) (**Figure 3A**).

**FIGURE 5:**
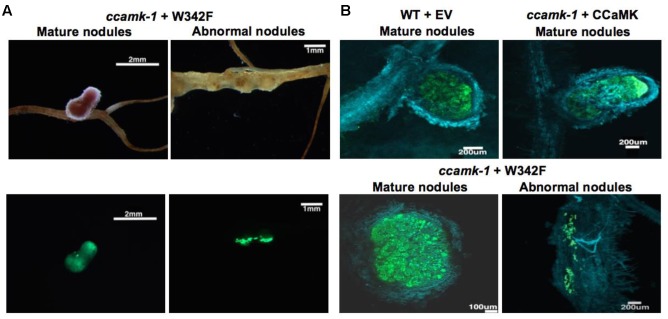
**(A)** Morphological differences in nodules produced on roots complemented with W342F and CCaMK; **(B)** Confocal microscopic images of mature nodules from roots of wild-type *Medicago truncatula* and *ccamk-1* knockout complemented with CCaMK and W342F mutant. Blue color indicates cell wall tissue stained with Calcofluor white. Green color shows living *S. meliloti* 2011.

Although the total number of nodules was equivalent between *ccamk-1* + CCaMK and *ccamk-1* + W342F plants, the number of mature, pink nodules on the *ccamk-1* + W342F plants roots was significantly less than that on the *ccamk-1* + CCaMK roots (*p*-value = 0.01). The *ccamk-1* + CCaMK roots produced 100% mature, pink nodules after inoculation with rhizobia. Approximately 50% of the nodules on the *ccamk-1* + W342F plants were small abnormal nodules. These small nodules were not observed in the wild-type CCaMK inoculated with rhizobia (*p*-value = 0.00069) (**Figure 3B**). The abnormal nodules were white, indicating poor colonization by rhizobia (**Figure 5A**). Confocal microscopic analysis revealed that GFP-labeled *S. meliloti* 2011 was inside these small nodules but the distribution patterns of the bacteria in the nodule differed from that of the normal mature nodules (**Figure 5B**).

## Discussion

Extensive studies have been conducted by using site-directed mutagenesis as a powerful tool to manipulate the coding region of various genes. We took advantage of this well-known strategy to modify a residue on the autoinhibitory/CaM-binding domain in order to alter its well-defined structure and modify the CaM-binding capacity of CCaMK. Altering the CaM-binding capacity of CCaMK should provide a better understanding of the regulation of CCaMK’s autoinhibitory domain.

The CaM-binding domain contains particular structural features: it forms an α helix with a net positive charge and two hydrophobic anchor residues on one side. These characteristics allow CaM to bind easily in the presence of Ca^2+^ (Yang et al., 2007). Typically, CaM contains two lobes at its N- and C-terminals that are connected by a helix linker, and in the presence of Ca^2+^, these two lobes will wrap the CaM-binding domain (Tidow and Nissen, 2013). However, there have been reports that some CaM-binding domains showed increased affinity to either of the terminals (Snedden and Fromm, 2001; Liu et al., 2012). This suggests that manipulating the α helix motif can modify its capacity to bind to CaM (Jang et al., 2011; Gifford et al., 2013).

One amino acid in the autoinhibitory/CaM-binding domain of CCaMK (W342) was replaced by amino acids with similar hydrophobicity but smaller masses in order to alter the α helix structure of the CaM-binding domain. Our data showed that reducing the mass of the selected amino acid could alter the capacity of CCaMK to interact with CaM. The mutant W342F showed an increase of CaM-binding in comparison to CCaMK, in addition, mutations W342L and W342I were able to interact with CaM but at lower levels. The mutant W342V did not interact with CaM. Interestingly, W342A mutant was able to bind to CaM similar to wild-type CCaMK even though its mass is relatively lower than that of the valine residue. Therefore, there is no direct correlation between mass of the residue at amino acid 342 and the CCaMK’s CaM-binding activity.

W342F mutant had an increase of CaM-binding in comparison to CCaMK. Hence, we postulated that the kinase activity of the mutant could be significantly affected. *In vitro* analysis of CCaMK revealed that autophosphorylation of wild-type CCaMK is Ca^2+^-dependent, and it is able to phosphorylate its substrate only in the presence of Ca^2+^ and CaM. In this study, we observed that W342F mutant was autophosphorylated and could phosphorylate MBP substrate in the absence of Ca^2+^ and CaM. This strongly suggests that the activity of W342F is Ca^2+^- and CaM-independent. Even though W342F was able to phosphorylate MBP *in vitro* in the presence of Ca^2+^ and CaM, but the mutant barely responded to Ca^2+^ and CaM stimulation, the intensity of maximum level of MBP phosphorylation was reduced in comparison to that catalyzed by wild-type CCaMK. These results clearly indicate that the CaM-binding domain is critical for the activation of CCaMK. Also, they suggest that CCaMK protein structure may be critical for its regulation because of the possible structural changes when Ca^2+^ and CaM bind. The proximity of W342 to two well-known autophosphorylation sites S343 and S344 might suggest that of Trp-342 could possibly play a role during the autophosphorylation of these two sites (Routray et al., 2013; Jauregui et al., 2017).

Our physiological results demonstrated that the W342F mutation has a negative effect on the nodule morphology, since a significant portion of the nodules observed in the *ccamk-1* + W342F plants were abnormal. These abnormal nodules were smaller and appeared to be infected with fewer rhizobia. This may correlate with the compromised substrate phosphorylation. The W342F mutant exhibited substrate phosphorylation in both the presence and absence of Ca^2+^ and CaM, but the intensity of the phosphorylation was less than that of the wild-type in the presence of Ca^2+^ and CaM. This suggests that even though this mutant could be activated in the absence and presence of Ca^2+^ or CaM, it may not be able to sufficiently phosphorylate the target protein in order to obtain a well-developed, mature nodule in the root. In addition, our mutants did not produce any spontaneous nodules in the absence of bacteria (data not shown). This suggests that enhancing autophosphorylation and substrate phosphorylation in the absence of Ca^2+^ and CaM does not result in an “autoactive” kinase that can stimulate a pathway toward spontaneous nodulation. Therefore, we hypothesize that a threshold of kinase activity must be reached and this could trigger the production of normal, mature nodules. Since our mutant showed some reduction in the intensity of substrate phosphorylation in presence of Ca^2+^ and CaM, this suggests that phosphorylation may not reach the putative threshold. Hence, the development of many nodules cannot reach the mature stage. Another possibility is that the stability of W342F mutation could be compromised when expressed in the roots.

This study documents that altering the autoinhibitory domain of CCaMK can significant increase the CaM-binding capacity of CCaMK which leads to a negative impact on root nodule development. Understanding of some target proteins of CCaMK involved in the regulation of nodule development might help to understand how CCaMK is involved not only for decoding Ca^2+^ oscillation but also in regulating some down-stream targets of root nodule symbiosis.

## Author Contributions

EJ conceived the idea for this project in consultation with BP and LD, performed most of the experiments, analyzed results and wrote most of the paper. LD and CG advised, helped in data analysis and supported with further ideas and experiments for the project. BP provided financial support, helped in planning, executing and leading the project. EJ, LD, CG, and BP were involved in manuscript preparation.

## Conflict of Interest Statement

The authors declare that the research was conducted in the absence of any commercial or financial relationships that could be construed as a potential conflict of interest.
