# Supplementary material for: W342F Mutation in CCaMK Enhances Its Affinity to Calmodulin But Compromises Its Role in Supporting Root Nodule Symbiosis in Medicago truncatula
Source: Front Plant Sci. 2017 Nov 16;8:1921. doi: 10.3389/fpls.2017.01921 (PMC5696362; doi:10.3389/fpls.2017.01921)
Supplement: Supplementary file 1 [file Image_1.PDF]

Supplemental Material

W342F mutation in CCaMK enhances its affinity to calmodulin but compromises its role in supporting root nodule symbiosis in *Medicago truncatula*

Edgard Jauregui[1], Liqun Du[1, 2], Cynthia Gleason[3] and B. W. Poovaiah[1*]

[1] Laboratory of Molecular Plant Science; Department of Horticulture; Washington State University; Pullman, WA, USA
[2] College of Life and Environmental Science; Hangzhou Normal University, China
[3] Department of Plant Pathology; Washington State University; Pullman, WA, USA

*Correspondence: B.W. Poovaiah, Poovaiah@wsu.edu.

1. Supplementary Figures

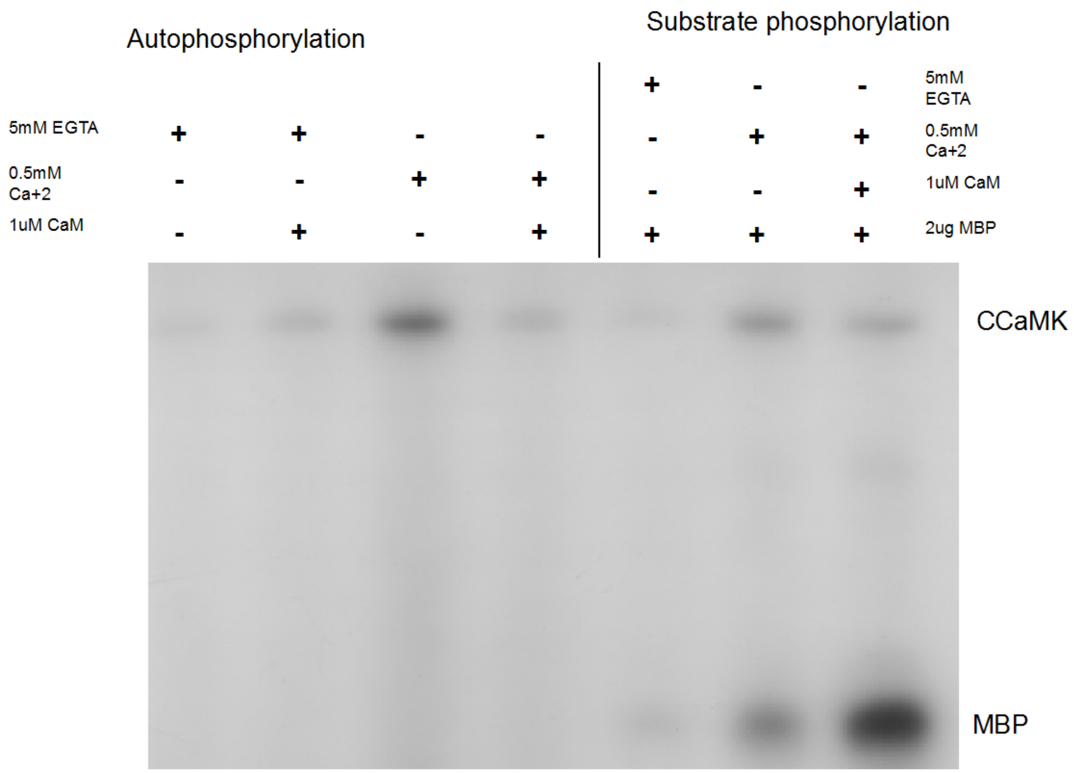

Supplemental Figure 1. Autoradiograph showing autophosphorylation and substrate phosphorylation. Autophosphorylation and substrate phosphorylation was carried out by using 0.4µg of purified CCaMK as described in material and methods section. See detailed description in Figure 2.

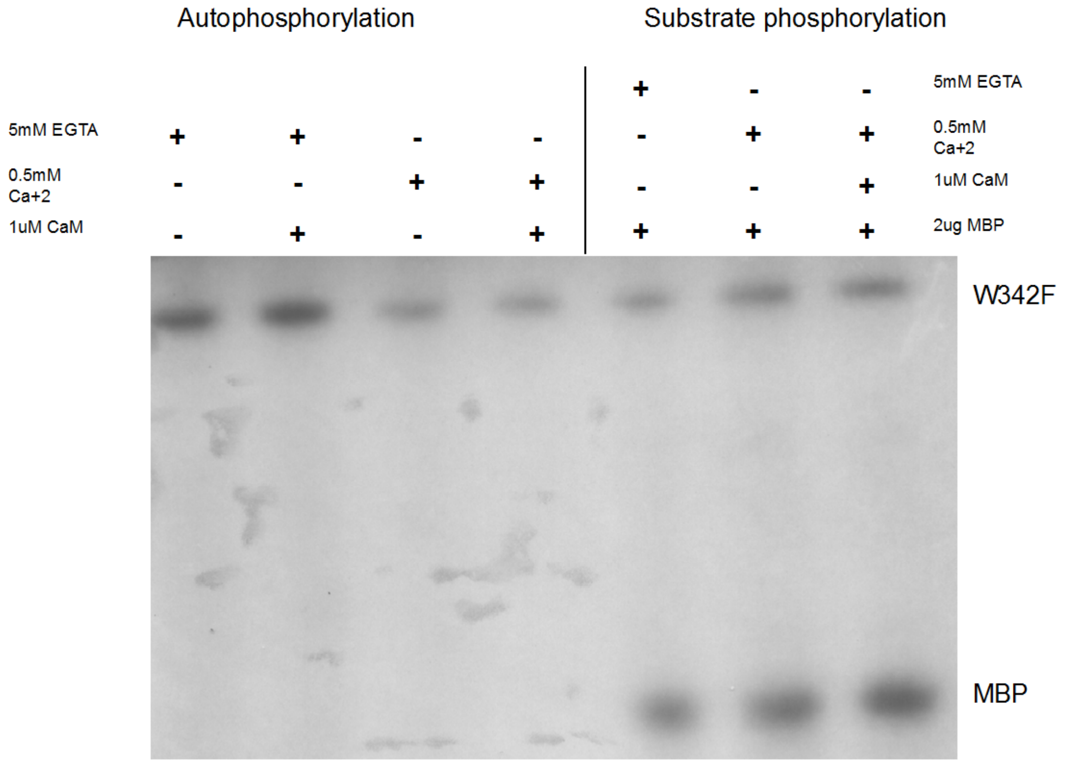

Supplemental Figure 2. Autoradiograph showing autophosphorylation and substrate phosphorylation. Kinase activity of W342F mutant was performed as described in material and method section and described in Figure 2.
